# The effects of environmental and genetic factors on the germination of basidiospores in the *Cryptococcus gattii* species complex

**DOI:** 10.1038/s41598-018-33679-2

**Published:** 2018-10-15

**Authors:** Man You, Jianping Xu

**Affiliations:** Department of Biology, McMaster University, 1280 Main St West, Hamilton, Ontario L8S 4K1 Canada

## Abstract

Natural and artificial hybridization has been frequently reported among divergent lineages within and between the two closely related human pathogenic fungi *Cryptococcus gattii* species complex and *Cryptococcus neoformans* species complex. However, the biological effects of such hybridization are not well known. Here we used five strains of the *C*. *neoformans* species complex and twelve strains of the *C*. *gattii* species complex to investigate the potential effects of selected environmental and genetic factors on the germination of their basidiospores from 29 crosses. We found that the germination rates varied widely among crosses and environmental conditions, ranging from 0% to 98%. Overall, the two examined media showed relatively little difference on spore germination while temperature effects were notable, with the high temperature (37 °C) having an overall deleterious effect on spore germination. Within the *C*. *gattii* species complex, one intra-lineage VGIII × VGIII cross had the highest germination rates among all crosses at all six tested environmental conditions. Our analyses indicate significant genetic, environmental, and genotype-environment interaction effects on the germination of basidiospores within the *C*. *gattii* species complex.

## Introduction

The genus *Cryptococcus* was created by Kützing in 1833^1^. This genus currently includes 37 species, two of which are well-known human fungal pathogens, the *Cryptococcus neoformans* species complex and the *Cryptococcus gattii* species complex^2,3^. Cryptococcosis caused by the *C*. *neoformans* species complex and *C*. *gattii* species complex is among the most serious fungal diseases and claims hundreds of thousands of lives each year, with an estimated global infection burden of 223,100 cases annually^4^. Current epidemiological studies suggest that the *C*. *neoformans* species complex infects primarily immunocompromised patients, whereas the *C*. *gattii* species complex is commonly found infecting immunocompetent individuals.

The *C*. *gattii* species complex was traditionally considered a pathogen associated with tropical and subtropical regions. However, over the last decade, strains of the *C*. *gattii* species complex has been commonly found in temperate regions of North America and Europe^5–11^. Recent molecular phylogenetic studies identified significant diversity and genetic divergence within both the *C*. *neoformans* species complex and the *C*. *gattii* species complex. In one study, the divergent lineages within each of the two species complexes were proposed as different species: the *C*. *neoformans* species complex was divided into two species (*C*. *neoformans* and *C*. *deneoformans*) and the *C*. *gattii* species complex into five species (*C*. *gattii*, *C*. *deuterogattii*, *C*. *bacillisporus*, *C*. *tetragattii* and *C*. *decagattii*) (Table 1). However, there is an ongoing debate about whether the observed differences warrant naming the different lineages as different species and on how species should be named in this and other groups of fungal taxa. Here we used the conservative naming system to call strains of lineages VNI to VNIV as belonging to the *C*. *neoformans* species complex while those of lineages VGI to VGIV as belonging to the *C*. *gattii* species complex. Regardless of the naming disagreements, these lineages/species are readily identified using a variety of molecular markers, including multi-locus sequence typing (MLST) and amplified fragment length polymorphism (AFLP) analysis (Table 1)^12,13^. Within the *C*. *gattii* species complex, Bovers *et al*. reported that VGI and VGIII are the most closely related, VGIV clusters basal to them, whereas VGII is the most distantly related to others. Within the *C*. *neoformans* species complex, VNI and VNII are sister groups, VNIV being the most distantly related, and VNIII representing the hybrids between VNI or VNII with VNIV^14^.

**Table 1:** Correspondence among the names that have been used to describe the various lineages within the human pathogenic *Cryptococcus* species complexes.

Name in this manuscript	Common/variety name	Lineage/Molecular type	Proposed name^12^
*Cryptococcus neoformans species complex*	*C*. *neoformans* var. *grubii*	VNI/VNII/VN (AFLP1, AFLP1A, AFLP1B, VNB)	*C*. *neoformans*
	*C*. *neoformans* var. *neoformans*	VNIV (AFLP2)	*C*. *deneoformans*
	AD hybrid	VNIII (AFLP3)	*C*. *neoformans* × *C*. *deneoformans* hybrid
*Cryptococcus gattii species complex*	*C*. *gattii*	VGI (AFLP4)	*C*. *gattii*
		VGII (AFLP6)	*C*. *deuterogattii*
		VGIII (AFLP5)	*C*. *bacillisporus*
		VGIV (AFLP7)	*C*. *tetragattii*
		VGIV/VGIIIc (AFLP10)	*C*. *decagattii*
Cryptococcal interspecific hybrids	DB hybrid	AFLP8	*C*. *deneoformans* × *C*. *gattii* hybrid
	AB hybrid	AFLP9	*C*. *neoformans* × *C*. *gattii* hybrid
	AB hybrid	AFLP11	*C*. *neoformans* × *C*. *deuterogattii* hybrid

Hybridization is defined as the process that leads to successful mating between individuals of genetically distinct populations or species, producing offspring of mixed genetic ancestry^15^. Hybridization and hybrids have been observed in animals and plants since antiquity, but the scientific study of hybrids didn’t begin until the mid-18^th^ century when Kölreuter showed that hybrid progeny often had intermediate phenotypes of the parents and that most of them were sterile^16^. Since then, hybridization has been continuously reported. However, the role of hybridization in long-term evolution has been debated and there are two opposite viewpoints among biologists. At one extreme, hybridization is regarded as a potent evolutionary force that creates opportunities for speciation and adaptive evolution. Biologists holding this view believe hybridization increases the genetic diversity and brings novel gene combinations, which could lead to significantly increased adaptive potential in heterogeneous environments. The contrary considers hybridization as evolutionary noise, with only transient effects on populations and relatively little long-term evolutionary importance^17,18^. Recent genetic and genomic evidence suggest that hybridization is very common and has likely played a significant role in speciation and other biological processes of many organisms^19–21^.

The impact of hybridization on fungal evolution differs from those in the majority of plants and animals in several aspects. For example, unlike in plants and animals, most fungal species can reproduce both sexually and asexually. As a result, sexually sterile hybrid progeny can continue to reproduce through asexual/clonal reproduction and thus can have continuous evolutionary effects. Another unique characteristic of fungi is that they generally have short reproductive cycles and can achieve large population sizes in a relatively short period of time. Both features can contribute to the possibility that hybrid progeny with high fitness could emerge during hybridization in fungi.

Although most studies of hybridization have focused on animals and plants, natural and artificial hybridization has been frequently reported in many microbial groups, including the human pathogenic *Cryptococcus*. Both the *C*. *neoformans* species complex and the *C*. *gattii* species complex have a bipolar mating system with two different mating types, *MAT***a** and *MAT*α. Under appropriate conditions, strains of different mating types from the same or different lineages and species complexes can mate with each other to generate meiotic progeny. The hybridization process includes cell fusion, formation of dikaryotic hyphae, and the generation of basidiospores through meiosis^22^. In addition, natural hybrid strains have been frequently reported in the human pathogenic *Cryptococcus* species. For example, strains of *C*. *neoformans* var. *grubii* × *C*. *neoformans* var. *neoformans* (or VNI × VNIV and VNII × VNIV hybrids) have been observed in both the natural environments and in patients^23^. Indeed, the frequency of *C*. *neoformans* var. *grubii* × *C*. *neoformans* var. *neoformans* hybrids is reported to be ~6% among global clinical isolates and 30% among European clinical isolates^24,25^. In addition to the *C*. *neoformans* var. *grubii* × *C*. *neoformans* var. *neoformans* hybrids, both inter-lineage and inter-species hybrids have been described in the *C*. *neoformans/C*. *gattii* species complex. Specifically, three *C*. *neoformans* var. *neoformans* VNIV × *C*. *gattii* VGI hybrids from two HIV-negative patients in the Netherlands, one *C*. *neoformans* var. *grubii* VNI × *C*. *gattii* VGI hybrid from an HIV-positive person in Canada, and one novel *C*. *neoformans* var. *grubii* VNI × *C*. *gattii* VGII hybrid from South America, have been reported^14,26–29^. In addition, Kavanaugh *et al*. reported an ancient introgression event that a fragment of the *C*. *neoformans* var. *grubii* gene region (~40 kb) non-reciprocally transferred to a strain of the *C*. *neoformans* var. *neoformans*^30^. These results suggest that hybridization is common in the human pathogenic *Cryptococcus* species complexes and that they represent great model organisms for understanding the effects of hybridization on fungal evolution.

One of the key indicators of evolution and speciation is the viability of sexual offspring. In the human pathogenic *Cryptococcus*, the sexual basidiospores are also considered the infectious propagules. These spores can be abundant, stress resistant, and readily aerially dispersed. As a result, animals may encounter spores more often than other infectious forms^31^. A previous study described that spores from the *C*. *neoformans* species complex were much more infectious than yeast cells in mice^32^. Both the *C*. *neoformans* species complex and the *C*. *gattii* species complex have defined sexual cycles that can produce abundant basidiospores capable of infecting patients^31^. However, based on the low spore germination rate at 37 °C, several studies also suggested that basidiospores might not be the only nor most important infectious propagule in *Cryptococcus*^33–35^.

A recent study reported that basidiospores from intra-lineage crosses within the *C*. *neoformans* species complex had an overall greater germination potential than those from inter-lineage crosses over a range of environmental conditions^33^. Their results showed that temperature had a greater influence than medium on spore germination, with lower germination at 37 °C than at 23 °C and 30 °C in most crosses. Whether a similar result will be found in the *C*. *gattii* species complex remains unknown. As described previously, the *C*. *neoformans* species complex and *C*. *gattii* species complex differ in several aspects, including geographic distributions, epidemiology, and mitochondrial inheritance^36,37^.

Here, we examined the germination of basidiospores from VGI × VGIII, VGII × VGIII, VGIII × VGIII crosses within the *C*. *gattii* species complex under selected environmental conditions. In addition, we performed several crosses between strains belonging to the two different species complexes *C*. *gattii* species complex and *C*. *neoformans* species complex. These results were compared with those reported previously for the *C*. *neoformans* species complex. We hypothesize that similar to what was observed in the *C*. *neoformans* species complex, basidiospores from the VGIII × VGIII crosses in the *C*. *gattii* species complex should show higher basidiospore germination rates than those of inter-lineage crosses. Furthermore, because *C*. *gattii* is mainly found in tropical and subtropical regions, we hypothesize that basidiospores from crosses within the *C*. *gattii* species complex should germinate well at a high temperature due to their historical adaptation.

## Results

In this study, we used a total of twelve strains of the *C*. *gattii* species complex and five strains of the *C*. *neoformans* species complex, including three VGI strains, three VGII strains, six VGIII strains, three VNI strains and two VNIV strains (Table 2). Among these 17 strains, eight were *MAT***a** and nine were *MAT*α. Fifteen of these strains were wildtype while two strains JF101 (*MAT*α) and JF109 (*MAT***a**) had mutations at the *crg1* gene^38^. The *crg1* gene is a suppressor of the mating pathway in *C*. *gattii* species complex and its knockout enabled these two strains to mate much more efficiently than wildtype strains. However, the knockout is not known to have other notable effects, including meiosis and sporulation^38^. These strains were used to create 46 mating pairs. For each successful cross, we examined the basidiospore germination rates on two media and at three temperatures. Below we describe the effects of the examined environmental and genetic factors on basidiospore germination among our mating crosses.

**Table 2:** Strains used in this study.

Strain	Lineage/Molecular Type (GenBank accession numbers for GPD1, LAC1, and PLB1)	Mating Type	Strain source
B4545	VGI (DQ096396; DQ096404; DQ096353)	*MAT* **a**	Clinical, USA
B4492	VGI (MG891763; MG891768; MG891773)	*MAT*α	Clinical, USA
B4495	VGI (MG891764; MG891769; MG891774)	*MAT* **a**	Clinical, Australia
LA55n	VGII (HM990744; AY973087; HM990885)	*MAT* **a**	Clinical, Brazil
LA61n	VGII (HM990746; AY973089; HM990887)	*MAT*α	Clinical, Brazil
R265	VGII (DQ096377; DQ096400; DQ096343)	*MAT*α	Clinical, Canada
B4544	VGIII (MG891765; MG891771; MG891776)	*MAT*α	Clinical, USA
B4546	VGIII (DQ096383; DQ096405; AY327616)	*MAT* **a**	Clinical, USA
B4499	VGIII (MG891766; MG891770; MG891775)	*MAT*α	Clinical, Australia
ATCC32608	VGIII (DQ096388; DQ096406; FJ705950)	*MAT* **a**	Clinical, USA
JF101	VGIII (DQ096378; FJ706051; AY327615)	*MAT*α	Lab, derivative of NIH312
JF109	VGIII (DQ096383; FJ706051; AY327616)	*MAT* **a**	Lab, derivative of B4546
CDC15	VNI (MG891767; MG891772; MG891777)	*MAT*α	Clinical, USA
KN99**a**	VNI (GU079851; EF211594; GU079701)	*MAT* **a**	Lab, derivative of H99
KN99α	VNI (GU079851; EF211594; GU079701)	*MAT*α	Lab, derivative of H99
JEC20**a**	VNIV (HQ851613; EF211637; EU408650)	*MAT* **a**	Lab
JEC21α	VNIV (HQ851614; EF211638; HQ851729)	*MAT*α	Lab

### Mating success

Among the forty-six attempted crosses, twenty-nine were successful (Table 3). Our results indicated that mating success differed among the different types of crosses among the tested strains. Among strains between VGI and VGIII lineages, six of the nine hybrid crosses mated successfully (~66.7%). Seven out of the nine hybrid crosses between strains of the VGII and VGIII lineages were successful (~77.8%). All nine VGIII × VGIII crosses mated successfully (100%) and all seven VGIII × VN (*Cryptococcus neoformans*) were successful (100%). Despite multiple tries, we were unable to successfully cross strains from within and between other lineages of the *C*. *gattii* species complex and *C*. *neoformans* species complex. These included two VGI × VGI, two VGII × VGII, five VGI × VGII, two VGI × VNI, and one VGII × VNI (Table 3).

**Table 3:** Mating success among the 46 attempted crosses in this study.

*MAT*a Strain			*MAT*α Strain		Successful on V8
**VGI** × **VGI**					
B4545	VGI	X	B4492	VGI	−
B4495	VGI	X	B4492	VGI	−
**VGI** × **VGII**					
B4545	VGI	X	LA61	VGII	−
B4545	VGI	X	R265	VGII	−
B4495	VGI	X	LA61	VGII	−
B4495	VGI	X	R265	VGII	−
**VGI** × **VGIII**					
B4545	VGI	X	B4544	VGIII	+
B4545	VGI	X	B4499	VGIII	+
B4545	VGI	X	JF101	VGIII	+
B4495	VGI	X	B4544	VGIII	+
B4495	VGI	X	B4499	VGIII	+
B4495	VGI	X	JF101	VGIII	+
**VGII** × **VGI**					
LA55n	VGII	X	B4492	VGI	−
**VGII** × **VGII**					
LA55n	VGII	X	LA61	VGII	−
LA55n	VGII	X	R265	VGII	−
**VGII** × **VGIII**					
LA55n	VGII	X	B4544	VGIII	−
LA55n	VGII	X	B4499	VGIII	−
LA55n	VGII	X	JF101	VGIII	+
**VGIII** × **VGI**					
B4546	VGIII	X	B4492	VGI	−
ATCC32608	VGIII	X	B4492	VGI	−
JF109	VGIII	X	B4492	VGI	−
**VGIII** × **VGII**					
B4546	VGIII	X	LA61	VGII	+
B4546	VGIII	X	R265	VGII	+
ATCC32608	VGIII	X	LA61	VGII	+
ATCC32608	VGIII	X	R265	VGII	+
JF109	VGIII	X	LA61	VGII	+
JF109	VGIII	X	R265	VGII	+
**VGIII** × **VGIII**					
B4546	VGIII	X	B4544	VGIII	+
B4546	VGIII	X	B4499	VGIII	+
B4546	VGIII	X	JF101	VGIII	+
ATCC32608	VGIII	X	B4544	VGIII	+
ATCC32608	VGIII	X	B4499	VGIII	+
ATCC32608	VGIII	X	JF101	VGIII	+
JF109	VGIII	X	B4544	VGIII	+
JF109	VGIII	X	B4499	VGIII	+
JF109	VGIII	X	JF101	VGIII	+
***C***. ***gattii*** × ***C***. ***neoformans***					
B4545	VGI	X	CDC15	VNI	−
B4495	VGI	X	CDC15	VNI	−
LA55n	VGII	X	CDC15	VNI	−
B4546	VGIII	X	CDC15	VNI	+
ATCC32608	VGIII	X	CDC15	VNI	+
JF109	VGIII	X	CDC15	VNI	+
JF109	VGIII	X	JEC21α	VNIV	+
JF109	VGIII	X	KN99α	VNI	+
JEC20a	VNIV	X	JF101	VGIII	+
KN99a	VNI	X	JF101	VGIII	+

‘−’: failed.

‘+’: succeeded.

Of the 29 successful crosses, all involved strains from VGIII and 15 of which involved either JF101 or JF109. However, a comparison of the crosses involving VGIII strains with or without the *crg1Δ* mutation showed that the high mating success rate for VGIII strains observed here was not due to the *crg1Δ* mutation in strains JF101 and JF109. Specifically, even though crosses involving JF101 and JF109 mated more readily and produced more hyphae (a signature of mating product for the *C*. *gattii* species complex and the *C*. *neoformans* species complex) than other crosses (data not shown), for strains within VGIII, there was no noticeable difference in mating success rate between wildtype strains and the two *crg1Δ* mutants JF101 and JF109. Indeed, excluding the 15 crosses involving JF101 and JF109 from our comparisons of mating success still showed that VGIII strains were more fertile than strains in other lineages examined in this study (Table 3).

### Basidiospore germination

The rates of basidiospore germination were examined from the twenty-nine successful crosses. The basidiospores were spread-plated on two different media (a nutrient-rich YEPD medium and a nutrient-limited SD medium) and incubated at three different temperatures (23 °C, 30 °C and 37 °C). These crosses were divided into four groups based on the lineage associations of the parental strains: VGI × VGIII, VGII × VGIII, VGIII × VGIII and VN × VGIII. The summary of basidiospore germination rates is shown in Table 4.

**Table 4:** Rates of basidiospore germination after 7 days of incubation for the 29 successful crosses under six environmental conditions. Values represent means ± SD of three independent tests.

Mating Cross	Medium	Temperature	Mean rates of Basidiospores Germinated (N = 300)
B4545 (VGI) × B4544 (VGIII)	SD	23 °C	9.67 ± 0.94
		30 °C	13.33 ± 0.47
		37 °C	1.67 ± 0.47
	YEPD	23 °C	11.00 ± 0.82
		30 °C	7.67 ± 1.25
		37 °C	3.33 ± 0.47
B4545 (VGI) × JF101(VGIII)	SD	23 °C	20.00 ± 3.56
		30 °C	28.67 ± 0.29
		37 °C	4.67 ± 0.94
	YEPD	23 °C	19.67 ± 4.50
		30 °C	26.67 ± 1.70
		37 °C	8.67 ± 1.25
B4495 (VGI) × B4544 (VGIII)	SD	23 °C	29.00 ± 4.32
		30 °C	27.33 ± 3.09
		37 °C	8.67 ± 2.05
	YEPD	23 °C	27.00 ± 5.72
		30 °C	25.67 ± 3.68
		37 °C	28.33 ± 2.49
B4495 (VGI) × JF101 (VGIII)	SD	23 °C	14.33 ± 3.68
		30 °C	12.00 ± 0.82
		37 °C	1.17 ± 0.47
	YEPD	23 °C	15.50 ± 4.32
		30 °C	13.00 ± 3.27
		37 °C	1.33 ± 1.25
B4545 (VGI) × B4499 (VGIII)	SD	23 °C	6.00 ± 3.74
		30 °C	7.67 ± 6.60
		37 °C	0.67 ± 1.25
	YEPD	23 °C	12.00 ± 0.82
		30 °C	5.83 ± 5.31
		37 °C	2.17 ± 1.25
B4495 (VGI) × B4499 (VGIII)	SD	23 °C	1.67 ± 0.47
		30 °C	1.17 ± 0.94
		37 °C	1.67 ± 0.47
	YEPD	23 °C	2.00 ± 0.00
		30 °C	3.17 ± 0.94
		37 °C	3.00 ± 3.56
LA55n (VGII) × JF101 (VGIII)	SD	23 °C	7.67 ± 2.36
		30 °C	6.33 ± 0.47
		37 °C	2.67 ± 0.94
	YEPD	23 °C	6.00 ± 2.16
		30 °C	3.67 ± 1.25
		37 °C	5.33 ± 0.47
B4546 (VGIII) × R265 (VGII)	SD	23 °C	13.33 ± 1.70
		30 °C	14.67 ± 1.70
		37 °C	12.67 ± 0.94
	YEPD	23 °C	17.33 ± 3.40
		30 °C	21.67 ± 2.87
		37 °C	13.67 ± 3.40
ATCC32608 (VGIII) × LA61n (VGII)	SD	23 °C	11.33 ± 2.05
		30 °C	3.67 ± 1.25
		37 °C	10.33 ± 1.70
	YEPD	23 °C	9.67 ± 0.94
		30 °C	19.00 ± 2.16
		37 °C	10.00 ± 1.41
ATCC32608 (VGIII) × R265 (VGII)	SD	23 °C	47.67 ± 6.80
		30 °C	53.67 ± 0.94
		37 °C	36.33 ± 3.68
	YEPD	23 °C	44.33 ± 1.25
		30 °C	55.00 ± 1.41
		37 °C	53.67 ± 1.25
JF109 (VGIII) × LA61n (VGII)	SD	23 °C	15.67 ± 4.71
		30 °C	9.33 ± 0.47
		37 °C	13.67 ± 3.09
	YEPD	23 °C	14.00 ± 2.83
		30 °C	13.00 ± 0.82
		37 °C	14.67 ± 2.49
JF109 (VGIII) × R265 (VGII)	SD	23 °C	38.67 ± 4.78
		30 °C	40.00 ± 5.72
		37 °C	30.33 ± 1.25
	YEPD	23 °C	31.67 ± 4.50
		30 °C	34.33 ± 5.31
		37 °C	30.00 ± 2.83
B4546 (VGIII) × LA61n (VGII)	SD	23 °C	11.00 ± 0.82
		30 °C	12.33 ± 1.25
		37 °C	11.67 ± 1.25
	YEPD	23 °C	16.33 ± 4.11
		30 °C	15.67 ± 4.78
		37 °C	15.33 ± 1.25
B4546 (VGIII) × B4544 (VGIII)	SD	23 °C	80.00 ± 4.55
		30 °C	86.67 ± 0.94
		37 °C	58.33 ± 2.36
	YEPD	23 °C	98.33 ± 0.47
		30 °C	89.67 ± 0.94
		37 °C	89.00 ± 4.32
B4546 (VGIII) × B4499 (VGIII)	SD	23 °C	42.33 ± 10.87
		30 °C	30.00 ± 0.00
		37 °C	25.33 ± 4.19
	YEPD	23 °C	30.33 ± 2.05
		30 °C	32.33 ± 8.18
		37 °C	27.00 ± 1.63
B4546 (VGIII) × JF101 (VGIII)	SD	23 °C	11.67 ± 2.87
		30 °C	11.33 ± 1.25
		37 °C	12.67 ± 2.62
	YEPD	23 °C	12.00 ± 0.00
		30 °C	5.33 ± 0.47
		37 °C	10.33 ± 1.25
ATCC32608 (VGIII) × B4544 (VGIII)	SD	23 °C	23.67 ± 4.19
		30 °C	27.00 ± 1.41
		37 °C	1.00 ± 0.82
	YEPD	23 °C	18.67 ± 1.70
		30 °C	23.67 ± 2.05
		37 °C	21.67 ± 0.47
ATCC32608 (VGIII) × B4499 (VGIII)	SD	23 °C	28.67 ± 1.70
		30 °C	31.67 ± 2.05
		37 °C	29.33 ± 4.11
	YEPD	23 °C	27.33 ± 6.13
		30 °C	35.00 ± 5.10
		37 °C	23.67 ± 0.47
ATCC32608 (VGIII) × JF101 (VGIII)	SD	23 °C	0.67 ± 0.94
		30 °C	0.67 ± 0.47
		37 °C	1.33 ± 1.25
	YEPD	23 °C	0.00 ± 0.00
		30 °C	0.33 ± 0.47
		37 °C	0.00 ± 0.00
JF109 (VGIII) × B4544 (VGIII)	SD	23 °C	15.33 ± 0.47
		30 °C	16.00 ± 2.83
		37 °C	13.00 ± 3.56
	YEPD	23 °C	17.67 ± 2.05
		30 °C	19.33 ± 2.05
		37 °C	22.33 ± 0.47
JF109 (VGIII) × B4499 (VGIII)	SD	23 °C	22.00 ± 2.45
		30 °C	29.67 ± 3.09
		37 °C	23.00 ± 2.16
	YEPD	23 °C	27.00 ± 4.55
		30 °C	26.00 ± 7.26
		37 °C	22.67 ± 3.77
JF109 (VGIII) × JF101 (VGIII)	SD	23 °C	1.00 ± 0.82
		30 °C	0.50 ± 0.00
		37 °C	1.50 ± 1.41
	YEPD	23 °C	1.33 ± 0.47
		30 °C	2.17 ± 0.47
		37 °C	2.00 ± 0.00
B4546 (VGIII) × CDC15 (VNI)	SD	23 °C	36.00 ± 2.94
		30 °C	42.00 ± 0.82
		37 °C	41.67 ± 0.94
	YEPD	23 °C	43.33 ± 4.19
		30 °C	39.33 ± 1.25
		37 °C	34.67 ± 1.70
ATCC32608 (VGIII) × CDC15 (VNI)	SD	23 °C	33.00 ± 5.66
		30 °C	29.33 ± 3.40
		37 °C	24.00 ± 2.94
	YEPD	23 °C	35.00 ± 8.60
		30 °C	29.00 ± 1.41
		37 °C	14.33 ± 4.50
JF109 (VGIII) × CDC15 (VNI)	SD	23 °C	3.67 ± 2.36
		30 °C	4.83 ± 2.87
		37 °C	3.17 ± 1.70
	YEPD	23 °C	4.00 ± 0.00
		30 °C	1.83 ± 1.25
		37 °C	1.00 ± 1.41
JF109 (VGIII) × KN99α (VNI)	SD	23 °C	1.33 ± 0.47
		30 °C	7.00 ± 0.82
		37 °C	1.17 ± 1.70
	YEPD	23 °C	1.83 ± 1.25
		30 °C	2.00 ± 0.00
		37 °C	1.83 ± 1.89
KN99a (VNI) × JF101 (VGIII)	SD	23 °C	1.33 ± 0.47
		30 °C	1.33 ± 0.47
		37 °C	3.67 ± 0.94
	YEPD	23 °C	2.67 ± 0.47
		30 °C	1.00 ± 0.82
		37 °C	0.67 ± 0.47
JF109 (VGIII) × JEC21 (VNIV)	SD	23 °C	3.00 ± 1.41
		30 °C	1.33 ± 0.47
		37 °C	1.00 ± 0.00
	YEPD	23 °C	1.67 ± 0.47
		30 °C	0.67 ± 0.47
		37 °C	0.00 ± 0.00
JEC20 (VNIV) × JF101 (VGIII)	SD	23 °C	15.67 ± 3.09
		30 °C	22.00 ± 4.55
		37 °C	15.00 ± 7.12
	YEPD	23 °C	20.67 ± 4.64
		30 °C	16.00 ± 0.82
		37 °C	14.00 ± 2.45

Our comparisons of the basidiospore germination rate differences between the mating groups indicated significant differences between several groups: VGI × VGIII vs. VGII × VGIII (p = 0.05), VGI × VGIII vs. VGIII × VGIII (p = 0.001), and VGIII × VGIII vs. VN × VGIII (p < 0.0001). In all these cases, crosses involving genetically closely related strains on average produced basidiospores with higher germination rates than those from crosses involving more distantly related parental strains (Table 4). However, separating the comparisons into six different environmental conditions and compared each pair separately, significant differences were only found between two groups VN × VGIII vs. VGIII × VGIII on SD medium at 30 °C and YEPD medium at 37 °C, where p-values were 0.0491 and 0.0342 respectively.

As shown previously, the deletion of *crg1* gene enhanced the fertility of strains JF101 and JF109 as compared to their ancestral strains NIH312 and B4546 respectively^37^. Here we compared crosses involving strains B4546 and its *crg1Δ* derivative JF109 to examine the influence of *crg1* gene on basidiospore germination. A total of four pairwise comparisons were made: between B4546 × R265 and JF109 × R265; between B4546 × LA61n and JF109 × LA61n; between B4546 × B4544 and JF109 × B4544; between B4546 × JF101 and JF109 × JF101; and between B4546 × CDC15 and JF109 × CDC15. The results showed that in three of the four pairwise comparisons, the crosses containing B4546 had significantly higher germination rates than those containing JF109, consistent with *crg1* gene playing a role in spore germination. However, in one of the four pairwise comparisons, B4546 × R265 and JF109 × R265, the germination rate involving JF109 was significantly higher than that of involving B4546 (p < 0.0001).

### Effects of temperature on spore germination

**Figure 1:**
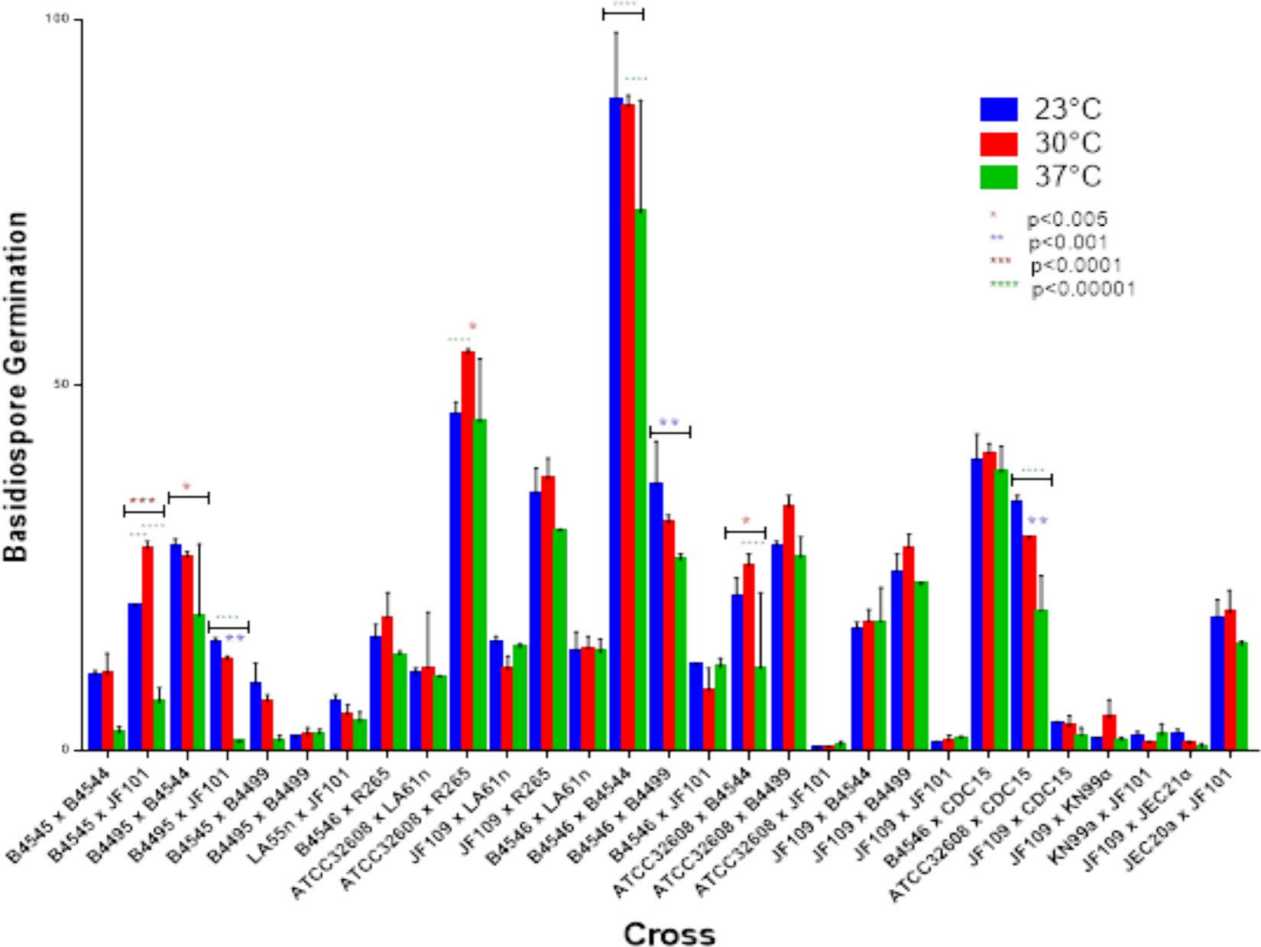
The mean rates of basidiospores germination at three different temperatures (23 °C, 30 °C and 37 °C). Values represent means ± SD.

Our results showed that temperature had a significant influence on basidiospore germination and that the effects were different for different crosses (Table 4; Fig. 1). The highest basidiospore germination rate was found from the intra-lineage cross between strains within VGIII lineage (B4546 × B4544) at all three temperatures, followed by an inter-lineage cross between VGII and VGIII strains (ATCC32608 × R265). In contrast, in two crosses, a VGIII intra-lineage cross (ATCC32608 × JF101, at 23 °C and 37 °C on YEPD) and an inter-species cross JF109 (VGIII) × JEC21 (VNIV) (at 37 °C on YEPD), there was no germination. Of the 29 successful crosses, three (two inter-lineage crosses ATCC32608 × LA61n and B4546 × LA61n, and one intra-lineage cross JF109 × B4544) showed minor differences in their spore germination rates among the three temperature treatments.

In general, among the three temperature environments, the 37 °C had the lowest germination rate for most crosses while the 23 °C and 30 °C temperature environments supported similar germination rates. However, there were some exceptions. For example, though statistically insignificant, seven crosses (KN99a × JF101; JF109 × JF101; JF109 × B4544; ATCC32607 × JF101; B4546 × JF101; JF109 × LA61n; and B4595 × B4499; Fig. 1) showed a comparable or higher germination rate at the 37 °C temperature than at the two lower temperatures. In addition, the spores in two inter-lineage crosses, ATCC32608 × R265 and B4545 × JF101, showed significantly higher germination rates at 30 °C than at 23 °C (p < 0.0001 in both cases). The detailed data and statistical comparisons among temperatures for each cross are shown in Table 4 and Fig. 1.

The above comparisons were based on basidiospore germination at seven days after incubation. We also attempted to obtain colony counts on day 2 and day 3 after incubation. At day 2, few basidiospores germinated to form visible colonies. By day 3, there were visible colonies for most crosses. The comparisons in colony counts between day 3 and 7 revealed that while most crosses showed similar spore germination between the two time-points in each of the six environmental conditions, several crosses did show significant differences (Data not shown). Specifically, the intra-lineage cross B4546 × B4544 (p < 0.001) at 23 °C, and the intra-lineage cross ATCC32608 × B4499 (p < 0.0001), the inter-lineage ATCC32608 × R265 (p < 0.0001) and the inter-species cross B4546 × CDC15 (p < 0.0001) at 37 °C on SD medium, all showed significant increases in basidiospore germination from day 3 to day 7. Overall, for most crosses, the fastest germination was observed at the 30 °C environment, followed by that at 37 °C and then at 23 °C (Data not shown).

### Effects of medium on spore germination

Compared to the large effects of temperature observed for many crosses, the effects of medium are overall relatively minor. However, there are several notable observations. First, two crosses, the intra-lineage cross ATCC32608 × JF101 (at 23 °C and 37 °C) and the inter-species cross JF109 × JEC21 (at 37 °C), showed no germinated basidiospores on the rich YEPD medium while germinations were observed on the minimal SD medium (Table 4). Second, significant differences between the two medium treatments were found for three crosses (inter-lineage cross B4546 × LA61n, intra-lineage crosses B4546 × B4499 and B4546 × B4544) at 23 °C, two crosses (inter-lineage cross ATCC32608 × LA61n and intra-lineage cross JF109 × B4499) at 30 °C, and six crosses (two inter-lineage crosses B4495 × B4544 and ATCC32608 × LA61n; three intra-lineage crosses B4546 × B4544, ATCC32608 × B4544 and JF109 × B4544; one inter-species cross B4546 × CDC15) at 37 °C with p < 0.001. In most of the above cases, the rich YEPD medium supported greater spore germination than the minimal SD medium. The exceptions were two crosses B4546 × CDC15 (at 37 °C) and JF109 × KN99α (at 30 °C) where the SD medium supported higher spore germination rates than the YEPD medium.

As indicated above, the media contributions to differences in germination rates from those crosses were temperature-specific. We thus further explored whether there was a broad temperature-medium interaction effect on basidiospore germination including all crosses and all environmental conditions. Using a generalized linear mixed model approach, we found no significant temperature-medium interaction effect on basidiospore germination in our crosses (Data not shown).

### Effect of genetic divergence between parental strains on their progeny basidiospore germination

**Figure 2:**
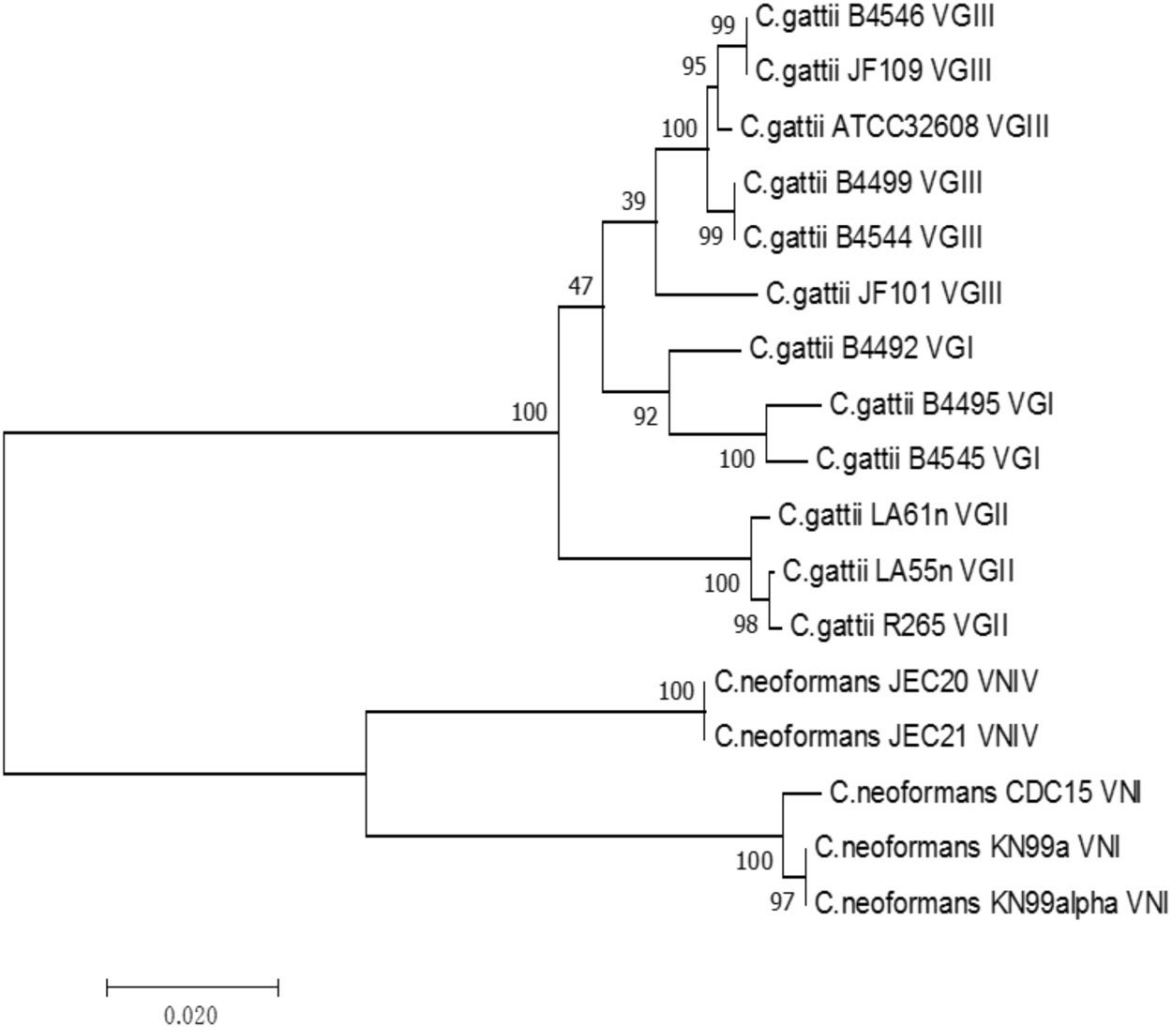
The genetic relationships among strains of the *Cryptococcus gattii* species complex and the *Cryptococcus neoformans* species complex analyzed in this study. The relationships were inferred based on DNA sequences at three gene fragments.

To examine the potential effects of nucleotide sequence divergence between parental strains on their progeny basidiospore germination, we obtained the DNA sequences at three gene fragments (*GPD*1, *LAC*1 and *PLB*1) for all parental strains. Of the 17 strains that resulted in successful crosses, 12 already had their sequences at these three loci deposited in GenBank and these were retrieved for our analyses. We obtained the sequences for the remaining five strains (B4492, B4495, B4544, B4499 and CDC15) using the method described previously. The sequence accession numbers of all the 51 sequences are presented in Table 2. A Neighbor-Joining tree based on the concatenated sequences was constructed using MEGA7 (Fig. 2). The phylogenetic tree showed the expected relationships among parental strains used in this study, with the strains from the same lineage grouped together. The Kimura-2-parameter genetic distances between each pair of mating parents were used to analyze its relationship with basidiospore germination rate.

The computed pairwise nucleotide sequence distances between parental strains are summarized in Supplementary Table S1. Based on the basidiospore germination data in Table 4 and the nucleotide sequence divergence data in Table S1, we conducted a series of correlation tests. These included each of the six environmental conditions and for different types of cross populations. The summary results are presented in Supplementary Table S2. Our correlation analyses showed that when all 29 crosses were included, there was a negative correlation between genetic distance of parental strains and the rate of basidiospore germination in all tested environments. This result is consistent with the hypothesis that sequence divergence contributes to lower progeny basidiospore viability. However, the correlations were statistically insignificant in any of the six individual environmental conditions (Supplementary Table S2).

To further explore the relationship between genetic distance and basidiospore germination rate, we divided the 29 crosses into four groups based on strain lineage relationships and examined the relationships between genetic distance and basidiospore germination rate within each type of crosses. These four groups were VGI × VGIII, VGII × VGIII, VGIII × VGIII, and VN × VGIII. Our analyses revealed that of the four groups, only the VGIII × VGIII group showed a slight negative relationship between genetic distance and spore germination rate (Supplementary Table S2). In contrast, the other three groups all showed slight positive correlations. However, none of the four correlations were statistically significant (p > 0.1).

## Discussion

In this study, we examined the germination rates of basidiospores from crosses involving strains from within and between diverged lineages in the *C*. *gattii* species complex and the *C*. *neoformans* species complex under six different environmental conditions. Interestingly, all our successful crosses involved at least one VGIII strain. This result is consistent with several previous studies that showed VGIII strains as being more fertile than strains of other VG groups^37–41^. Among the successful crosses, we found large variations in germination ability among crosses and among the tested environmental conditions. The basidiospore germination rates of an intra-VGIII lineage cross B4546 × B4544 were higher (average of ~84% and range from 58.33% to 98.33% among the six conditions) than other crosses. While media showed an overall relatively minor effects, temperature showed a significant influence on basidiospore germination, with the high temperature (37 °C) having an inhibitory effect for spores from many of the crosses. Below we discuss the spore germination rates observed here and compare with those in the *C*. *neoformans* species complex.

### Comparison of spore germination rates with those in the *C*. *neoformans* species complex

Previous studies have estimated the germination rates of basidiospores from crosses in the the *C*. *neoformans* species complex, with a range of 5.5%^42^ to 95%^33^. The highest rate of basidiospore germination in the *C*. *neoformans* species complex was found from an intra-lineage cross between two isogenic strains JEC20a × JEC21α belonging to the VNIV lineage on the minimal SD medium at 30 °C (~95%) and 23 °C (~90%), followed by two other intra-specific crosses KN99a × KN99α (~70%) and KN99a × CDC15 (~60%) belonging to the VNI lineage at 23 °C on SD medium. The lowest rate in that study was found in certain hybrid crosses between strains of VNI and VNIV. Our results showed that basidiospores from different crosses in the *C*. *gattii* species complex could also have highly variable germination rates, from 0% to 98%. The 0% germination was observed in two crosses: an inter-specific cross between VGIII and VNIV (JF109 × JEC21α) and an intra-lineage cross within VGIII (ATCC32608 × JF101) when their spores were placed under certain conditions. The highest germination rate was found in one of the intra-lineage crosses (VGIII, B4546 × B4544) at 23 °C (~98%), 30 °C (~90%) and 37 °C (89%) on YEPD medium. However, the second highest was an inter-lineage cross (VGIII × VGII, ATCC32608 × R265) at 30 °C (~55%) and 37 °C (~54%) on the YEPD medium. Similar to that observed in *C*. *neoformans*^33^, the germination rates of basidiospores from most crosses were less than 50%. Overall, lower rates of spore germination were found in the VGII × VGIII mating crosses. This observation is similar to that by Voelz *et al*. showing that both the VGIIa × VGIIIα and VGIIα × VGIIIa mating pairs had a low spore germination rate^43^. At present, the reason(s) for the overall lower germination rate of spores from VGII ×  × VGIII crosses remains unknown.

### Effects of environmental factors on spore germination and their implications

This study examined whether environmental factors (3 temperatures × 2 media) influenced the germination potential of basidiospores from hybrid crosses in *C*. *gattii* species complex. For human fungal pathogens, one of the most important characteristics is the ability to survive and grow at high temperatures (≥37 °C). However, for most crosses, our results demonstrated that high temperature had an inhibitory effect on basidiospore germination. This result is similar to what was found for basidiospores in all six crosses among strains within the *C*. *neoformans* species complex reported by Forsythe *et al*.^33^. Interestingly, different from what was found in *C*. *neoformans* species complex where the same two media did not show any noticeable effect on spore germination^33^, for several of the crosses examined here, media showed a significant effect on basidiospore germination, especially at 37 °C. For example, the rich YEPD medium supported a greater germination rate for 17/29 crosses than the minimum SD medium at both 23 °C and 37 °C (Table 4). The greater germination rate on YEPD medium than on SD medium for the *C*. *gattii* species complex spores may suggest that higher nutrient levels are more conducive for *C*. *gattii* species complex spore germination at low (23 °C) and high (37 °C) temperature rather than at intermediate (30 °C) temperature.

At present, the reasons for the diverse basidiospore germination rates among the different environmental conditions and among crosses are not known. One potential explanation might be related to mitochondrial inheritance. Previous studies have shown that different crosses in the *C*. *gattii* species complex showed very different mitochondrial inheritance patterns and that environmental factors such as temperature and UV irradiation can have significant effects on mitochondrial inheritance in both the *C*. *neoformans* species complex and the *C*. *gattii* species complex but to different degrees^37,44^. In general, Wang *et al*. noted that crosses among strains within the *C*. *gattii* species complex have more variable mtDNA inheritance patterns than those within the *C*. *neoformans* species complex and that parental strains, strain combinations, and diverse environmental factors can all contribute to the different mtDNA inheritance patterns in the *C*. *gattii* species complex^37^. The heterogeneous mitochondrial genotypes, including recombination genotypes, among basidiospore progeny may influence their energy generation and spore viability.

### Genetic contributions

Since all our successful matings involved at least one strain from the VGIII lineage, the specific characteristics of VGIII isolates might also have played a key role in the variable spore germinations observed here. Previous studies have shown that the VGIII lineage is genotypically highly diverse^45^ and that strains of both *MAT***a** and *MAT*α have been reported from clinical, veterinary and environmental VGIII isolates, consistent with sexual reproduction and recombination in natural populations of this lineage^46,47^. Frequent mating and recombination of these strains in nature could help explain the high mating success and high spore germination rates of the VGIII × VGIII crosses.

Our results indicate that c*rg1* gene plays a role on basidiospore germination with the effects differ among crosses. However, since not all crosses were similarly affected, the effects of the *crg1* mutation on basidiospore germination likely involve interacting with other gene(s) in the mating partner genome that likely differ among the parental strains. Several studies have reported that the deletion of *crg1* gene can enhance mating efficiency in both the *C*. *neoformans* species complex and the *C*. *gattii* species complex but may reduce the viability of basidiospores^38,43,48,49^, though no directional effect of the *crg1* gene on mitochondrial inheritance in *C*. *gattii* was found^50^. Voelz *et al*. used VGIII strains carrying the *crg1*::*NEO* mutation mated with a VGII strain and found none of the dissected 63 basidiospores were viable^43^. Among our crosses, two (ATCC32608 × JF101 and JF109 × JEC20) showed 0% of basidiospore germination rate under certain conditions. Of the four crosses where direct comparisons could be made about the role of *crg1* gene in basidiospore germination, three crosses showed significantly reduced spore viability when *crg1* was deleted while one cross showed the opposite. The results suggest that the effect of *crg1* gene on basidiospore germination is mating partner and incubation condition-dependent. Interestingly, strain R265 that showed a higher germination rate when mated with JF109 than when mated with B4546 represents the dominant genotype responsible for the cryptococcosis outbreak in western North America including British Columbia in Canada and Washington and Oregon States in the US. The potential ecological significance of this observation remains to be investigated.

Overall, our results suggested that nucleotide sequence divergence between two parental strains may be related to their progeny basidiospore germination. However, this result should be interpreted with caution. A previous study used the Cross-Match analysis revealed that the nucleotide sequence divergence between the VGI (WM276) and VGII (R265) genomes was ~7.6%, while that between VNI and VNIV was ~10%^51^. The similarity of concatenated sequences based on the consensus loci for multi-locus sequence typing (MLST) is 95% to 96% among the lineages of the *C*. *gattii* species complex and 84% to 86% between the *C*. *neoformans* species complex and the *C*. *gattii* species complex^14^. The average sequence divergence between mating pairs based on the three concatenated loci (*GPD*1, *LAC*1 and *PLB*1) used in this study was 1.1% for VGIII × VGIII crosses, 4.05% for VGI × VGIII crosses, 4.74% for VGII × VGIII crosses and 17.7% for VN × VGIII crosses. Thus, though there were some minor differences, overall, the amounts of sequence divergences among lineages within and between the *C*. *neoformans* species complex and the *C*. *gattii* species complex estimated using the different methods were similar to each other. As expected, there was an overall negative correlation between genetic divergence and germination rate when all crosses were included, consistent with a previous study reported for the *C*. *neoformans* species complex^33^. However, the observed correlation was not statistically significant in the present study. This was not surprising as there were large variations among crosses within each of the four mating categories analyzed here (Table 4 and Fig. 1). To eliminate some of the confounding factors associated with both parental strains being different in the comparison, we further assessed the relationship between genetic distance and basidiospore germination rate for crosses all involving a shared mating partner. Here, we focused on JF101, the only strain that successfully mated with representative strains from all lineages (VNI, VNIV, VGI, VGII, and VGIII) in this study. A total of eight crosses involving strain JF101 were included in this analysis (Tables 3 and 4). Correlational analyses between the pairwise parental strain genetic distances (Supplementary Table S1) and basidiospore germination rates (Table 4) in each of the six conditions did not yield any statistically significant correlation (p values all greater than 0.5). Overall, the results suggest that in the crosses examined here, the level of genetic divergence between parental strains is a relatively minor contributor to differences in basidiospore germination rate. In contrast, other factors such as genome structure differences (e.g. translocations and inversions) and mutations in genes involved in basidiospore germination likely play important roles. At present, almost nothing is known about the potential genome structural differences and mutations among the majority of investigated parental strains in this study.

Overall, our study identified that multiple factors can influence basidiospore germination rate differences, including parental strains/strain combination, temperature, medium, and some combinations of these factors. At present, the genetic bases for the observed differences are largely unknown. A previous study identified evidence for Bateson-Dobzjansky-Muller (BDM) incompatibility factors affecting the viability of basidiospores in a hybrid cross in the *C*. *neoformans* species complex^52^. In addition, several studies have also shown evidence that hybrids are unable to go through normal meiosis to generate haploid basidiospore progeny^13,41,42,52–55^. Both of which could contribute to genetic abnormality and low spore viability. However, this and earlier analyses have also shown that certain hybrid crosses between genetically divergent strains can generate highly viable spores with some showing superior phenotypic traits such as much faster growth rates and higher resistance to environmental stresses and antifungal drugs than parental strains^33,55,56^. In this study, we showed that novel hybrids could be readily generated in the laboratory among the divergent lineages within the *C*. *gattii* species complex and between strains of the *C*. *gattii* species complex and the *C*. *neoformans* species complex.

## Conclusions

This study described the potential environmental and genetic factors influencing the germination of basidiospores from among twenty-nine crosses within the *C*. *gattii* species complex and between the *C*. *gattii* species complex and the *C*. *neoformans* species complex. We examined the effects of two media, three temperatures, and genetic divergence between pairs of parental strains on the germination rates of basidiospores. Our analyses indicated that all examined factors (temperature, medium, parental strain and strain pair) could influence basidiospore germination. Unlikely in *C*. *neoformans* species complex where nucleotide sequence divergence between parental strains was negatively correlated with basidiospore germination rate, the results from the *C*. *gattii* species complex were more variable and complex. The highest spore germination rate was found in an intra-lineage VGIII × VGIII cross. In addition, while environmental factors can significantly influence the pattern of basidiospore germination, most of the environmental influences are not universal but are cross-specific. Our results also suggest that novel hybrids among certain lineages within the *C*. *gattii* species complex and between the *C*. *gattii* species complex and the *C*. *neoformans* species complex could be readily generated under laboratory conditions. The genotypic and phenotypic consequences of these hybridizations and their hybrids await further investigations.

## Materials and Methods

### Strains

Twelve strains of the *Cryptococcus gattii* species complex and five strains of the *Cryptococcus neoformans* species complex were used in this study. The twelve *C*. *gattii* species complex strains belonged to three lineages: VGI, VGII and VGIII. Strains B4545, B4492 and B4495 belong to the VGI lineage; strains LA55n, LA61n and R265 belong to the VGII lineage; and strains B4544, B4546, B4499, ATCC32608, JF101 and JF109 belong to the VGIII lineage. Strain JF109 is a derivative of a clinical strain B4546 with the *crg1* gene deleted while strain JF101 is derived from a clinical strain NIH312 with the deletion of *crg1* gene. Four of the five *C*. *neoformans* species complex strains correspond to two pairs of isogenic isolates, one pair is JEC20 and JEC21, the other one is KN99a and KN99α. The isogenic strain pairs differ at the mating type locus but are otherwise identical. CDC15 is a clinical isolate of the VNI lineage of the *C*. *neoformans* species complex. Strains B4544, B4492, B4499, LA61n, JF101, R265, JEC20a and KN99a are of mating type **a** while the remaining nine strains have the α mating type. The information of the parental strains used for mating crosses in this study is shown in Table 2.

### Mating and germination of basidiospores

The main objective of this study was to examine the rate of basidiospore germination for crosses involving strains within and among lineages of the *C*. *gattii* species complex. Not all 72 pairwise strain combinations between the *MAT***a** and *MAT*α types were crossed with each other. Instead, 46 pairwise combinations, including all 36 possible pairs within the *C*. *gattii* species complex were attempted (Table 3). To prepare for mating, all parental strains were first cultured on Yeast Extract-Peptone-Dextrose (YEPD) agar medium for 2–3 days at 30 °C. Actively growing cells were then re-suspended in sterile distilled water and 10 μl of the adjusted cell suspension (~10^5^ cells/ml) from each parental strain was completely mixed together and then spotted on the V8-juice mating agar medium. Each plate contained six spots (10 μl/spot): four for the mixed cells and two for pure parental cells as negative controls. Mating plates were incubated in the dark at room temperature (around 23 °C) for 7–30 days to allow for mating and sexual spore formation. Successful mating was indicated by the formation of hyphae at the periphery of the mating spots containing yeast cells from the two parents.

For those pairs that failed to mate at the first try, additional attempts were made, including by changing the pH of V8-juice agar medium, from pH7 to pH5. For all successful crosses, sections of agar with hyphae and basidiospores (i.e. no parental yeast cells) were cut and transferred to a new blank plate. The hyphae and basidiospores were gently washed by applying sterile 0.5% Tween 20 solution to the mycelial surface of each agar block and four layers of sterile cheesecloth (Loblaws Inc. Toronto, Canada) were used as filter to obtain pure spore solutions. Basidiospore suspensions were diluted with additional sterile 0.5% Tween 20 solution to a final density (approx. 3 × 10^3^ spores/ml). 100 μl of the diluted basidiospore suspensions was spread on either the common rich medium (YEPD agar medium) or the minimal Synthetic Dextrose (SD) agar medium and incubated at each of the tested temperatures (23 °C, 30 °C and 37 °C) for seven days. The number of all visible colonies formed by germinated basidiospores was counted on each plate at three and seven days respectively after incubation. For each cross × temperature × medium combination, we performed at least three repeats. The germination of basidiospores was determined as a ratio of the number of observed colonies to the estimated total number of basidiospores plated.

### Sequencing

To estimate the genetic divergence among parental strains, sequences in fragments of three protein-coding genes (*GPD*1, *LAC*1 and *PLB*1) were obtained from either the GenBank or through PCR followed by sequencing. For sequences of the strains that have already been deposited in the GenBank, their sequences were directly retrieved from the GenBank database. Those that were not found in the GenBank were obtained by PCR and sequencing of PCR products. Briefly, DNA was extracted from the parental strains following protocol outlined in Xu *et al*.^57^. The diluted DNA extraction was used as template to amplify the three gene fragments, following the PCR conditions listed in Supplementary Table S3. The amplificated PCR products were checked on a 1.2% agarose gel in 1x Tris-Acetic-Acid-EDTA buffer. The PCR products were then sequenced by MOBIX Lab at McMaster University (Hamilton, Ontario). The obtained sequences were edited using FinchTV 1.4.0 (Geospiza, Inc.; Seattle, WA, USA; http://www.geospiza.com) and aligned using MEGA 7.0 before being combined for phylogenetic analyses using the Neighbor-joining program. Bootstrap values were computed using 1000 replicates. In addition, the pairwise strain genetic distances were computed using MEGA 7.0. The relationship between genetic distances between mating partners and the basidiospore germination rates of the crosses was analyzed using a Pearson correlation test through the GraphPad Prism program (version 7.0; GraphPad Software, San Diego CA, USA).

### Statistical analyses

To compare the differences of basidiospore germination rate among crosses on tested conditions and the effects of environmental and genetic factors which contribute to the differences, we used the paired T-tests, the generalized linear mixed model using R^58^, and Pearson’s correlation and visualization using GraphPad Prism (version 7.0; GraphPad Software, San Diego CA, USA). P < 0.05 was considered statistically significant. Sequences alignment, genetic distance calculations and phylogenetic tree construction were performed using MEGA 7.0^59,60^.

### Statements on study approvals

We confirm that all methods in this study were carried out in accordance with relevant guidelines and regulations. In addition, all experimental protocols were approved by McMaster University.

## Electronic supplementary material

Supplementary Tables

## Data Availability

The new DNA sequences obtained and described in this study have been deposited in GenBank (MG891763 – MG891777).
