# Supplementary material for: The effects of environmental and genetic factors on the germination of basidiospores in the Cryptococcus gattii species complex
Source: Sci Rep. 2018 Oct 15;8:15260. doi: 10.1038/s41598-018-33679-2 (PMC6189041; doi:10.1038/s41598-018-33679-2)
Supplement: Supplementary file 1 — Supplementary Tables [file 41598_2018_33679_MOESM1_ESM.docx]

**Supplementary File for the Following Manuscript**

**Manuscript Title:** The effects of environmental and genetic factors on the germination of basidiospores in the *Cryptococcus gattii* species complex

**Author list:** Man You and Jianping Xu*

*Corresponding Address: Department of Biology, McMaster University, 1280 Main St West, Hamilton, Ontario, L8S 4K1, Canada. Email: jpxu@mcmaster.ca

Supplementary Table S1: The genetic distances between parental strains of successful crosses. Distances were calculated using the Kimura-2-Parameter model.

| Strain 1 | Strain 2 | Mean Genetic Distance | Standard Error |
| --- | --- | --- | --- |
| *C. gattii* B4545 VGI | *C. gattii* B4544 VGIII | 0.036 | 0.007 |
| *C. gattii* B4545 VGI | *C. gattii* JF101 VGIII | 0.046 | 0.008 |
| *C. gattii* B4495 VGI | *C. gattii* B4544 VGIII | 0.038 | 0.007 |
| *C. gattii* B4495 VGI | *C. gattii* JF101 VGIII | 0.049 | 0.008 |
| *C. gattii* B4545 VGI | *C. gattii* B4499 VGIII | 0.036 | 0.007 |
| *C. gattii* B4495 VGI | *C. gattii* B4499 VGIII | 0.038 | 0.007 |
| *C. gattii* LA55n VGII | *C. gattii* JF101 VGIII | 0.031 | 0.005 |
| *C. gattii* R265 VGII | *C. gattii* B4546 VGIII | 0.052 | 0.008 |
| *C. gattii* LA61n VGII | *C. gattii* ATCC32608 VGIII | 0.048 | 0.007 |
| *C. gattii* R265 VGII | *C. gattii* ATCC32608 VGIII | 0.049 | 0.008 |
| *C. gattii* LA61n VGII | *C. gattii* JF109 VGIII | 0.050 | 0.008 |
| *C. gattii* R265 VGII | *C. gattii* JF109 VGIII | 0.052 | 0.008 |
| *C. gattii* LA61n VGII | *C. gattii* B4546 VGIII | 0.050 | 0.008 |
| *C. gattii* B4544 VGIII | *C. gattii* B4546 VGIII | 0.008 | 0.002 |
| *C. gattii* B4499 VGIII | *C. gattii* B4546 VGIII | 0.008 | 0.002 |
| *C. gattii* B4546 VGIII | *C. gattii* JF101 VGIII | 0.014 | 0.004 |
| *C. gattii* ATCC32608 VGIII | *C. gattii* B4544 VGIII | 0.006 | 0.002 |
| *C. gattii* ATCC32608 VGIII | *C. gattii* B4499 VGIII | 0.006 | 0.002 |
| *C. gattii* ATCC32608 VGIII | *C. gattii* JF101 VGIII | 0.020 | 0.004 |
| *C. gattii* B4544 VGIII | *C. gattii* JF109 VGIII | 0.008 | 0.002 |
| *C. gattii* B4499 VGIII | *C. gattii* JF109 VGIII | 0.008 | 0.002 |
| *C. gattii* JF101 VGIII | *C. gattii* JF109 VGIII | 0.021 | 0.004 |
| *C. gattii* B4546 VGIII | *C. neoformans* CDC15 VNI | 0.182 | 0.022 |
| *C. gattii* ATCC32608 VGIII | *C. neoformans* CDC15 VNI | 0.180 | 0.022 |
| *C. gattii* JF109 VGIII | *C. neoformans* CDC15 VNI | 0.182 | 0.022 |
| *C. gattii* JF109 VGIII | *C. neoformans* KN99α VNI | 0.178 | 0.022 |
| *C. gattii* JF101 VGIII | *C. neoformans* KN99a VNI | 0.178 | 0.022 |
| *C. gattii* JF109 VGIII | *C. neoformans* JEC21 VNIV | 0.170 | 0.021 |
| *C. gattii* JF101 VGIII | *C. neoformans* JEC20 VNIV | 0.169 | 0.021 |

Supplementary Table S2: The Pearson correlation coefficient between environmental factors and the genetic distance.

| Group | | Genetic distance vs. YEPD (23^o^C) | Genetic distance vs. YEPD (30^o^C) | Genetic distance vs. YEPD (37^o^C) | Genetic distance vs. SD (23^o^C) | Genetic distance vs. SD (30^o^C) | Genetic distance vs. SD (37^o^C) |
| --- | --- | --- | --- | --- | --- | --- | --- |
| Mean Rates  (N=29) | Pearson r | -0.16 | -0.2263 | -0.2721 | -0.2136 | -0.1661 | -0.09351 |
|  | P-value (two-tailed) | 0.4071 | 0.2377 | 0.1533 | 0.2659 | 0.3891 | 0.6295 |
| VGI x VGIII  (N=6) | Pearson r | 0.3257 | 0.6088 | -0.08069 | 0.3546 | 0.5353 | 0.1261 |
|  | P-value (two-tailed) | 0.5287 | 0.1996 | 0.8792 | 0.4903 | 0.2738 | 0.8118 |
| VGII x VGIII  (N=7) | Pearson r | 0.4839 | 0.5244 | 0.4154 | 0.3928 | 0.3504 | 0.5457 |
|  | P-value (two-tailed) | 0.2712 | 0.2269 | 0.354 | 0.3834 | 0.441 | 0.2051 |
| VGIII x VGIII  (N=9) | Pearson r | -0.4775 | -0.5511 | -0.5042 | -0.5516 | -0.551 | -0.4782 |
|  | P-value (two-tailed) | 0.1936 | 0.1241 | 0.1663 | 0.1237 | 0.1241 | 0.1929 |
| VN x VGIII  (N=7) | Pearson r | 0.3654 | 0.3831 | 0.3165 | 0.3831 | 0.3509 | 0.3997 |
|  | P-value (two-tailed) | 0.4202 | 0.3963 | 0.4892 | 0.3963 | 0.4403 | 0.3744 |

Supplementary Table S3: Primers and PCR protocols for obtaining sequences of three genes. For all sets of primers the following PCR protocols were used.

| **Gene locus** | **Gene product** | **Primer Sequences (5’-3’)** | **Amplification conditions** | **Fragment size (bp)** | **Ref.** |
| --- | --- | --- | --- | --- | --- |
| **GPD1** | Glyceraldehyde-3-phosphate dehydroenase | **GPD1F:** CCACCGAACCCTTCTAGGATA **GPD1R:** CTTCTTGGCACCTCCCTTGAG | 94^o^C 3min; 35 cycles: 94^o^C 45s, 63^o^C 1min, 72^o^C 2min | 543 | 61 |
|  |  | **Alternative:**  **GPD1F:** TAGCGTTAGTACTAAACGAG  **GPD1R:** GTATTCGGCACCAGCCTCA | 12 cycles 62-56^o^C, step down 2^o^C every 2 cycles | 222 | 62 |
| **LAC1** | Laccase | **LAC1F:** AACATGTTCCCTGGGCCTGTG **LAC1R:** ATGAGAATTGAA TCGCCTTGT | 94^o^C 3min; 35 cycles: 94^o^C 30s, 53^o^C 30s, 72^o^C 1min | 469 | 61 |
|  |  | **Alternative:**  **LAC1F:** GGCGATACTATTATCGTA **LAC1R:** TTCTGGAGTGGCTAGAGC | 94^o^C 3min; 35 cycles: 94^o^C 1min, 50^o^C 1min, 72^o^C 1min | 565 | 14 |
| **PLB1** | Phospholipase | **PLB1F:** CTTCAGGCGGAGAGAGGTTT **PLB1R:** GATTTGGCGTTGGTTTCAGT | 94^o^C 3min; 35 cycles: 94^o^C 45s, 58^o^C 45s, 72^o^C 1min | 532 | 61 |
